# Supplementary material for: Oestrogen receptor α AF-1 and AF-2 domains have cell population-specific functions in the mammary epithelium
Source: Nat Commun. 2018 Nov 9;9:4723. doi: 10.1038/s41467-018-07175-0 (PMC6226531; doi:10.1038/s41467-018-07175-0)
Supplement: Supplementary file 1 — Supplementary Information [file 41467_2018_7175_MOESM1_ESM.pdf]

1

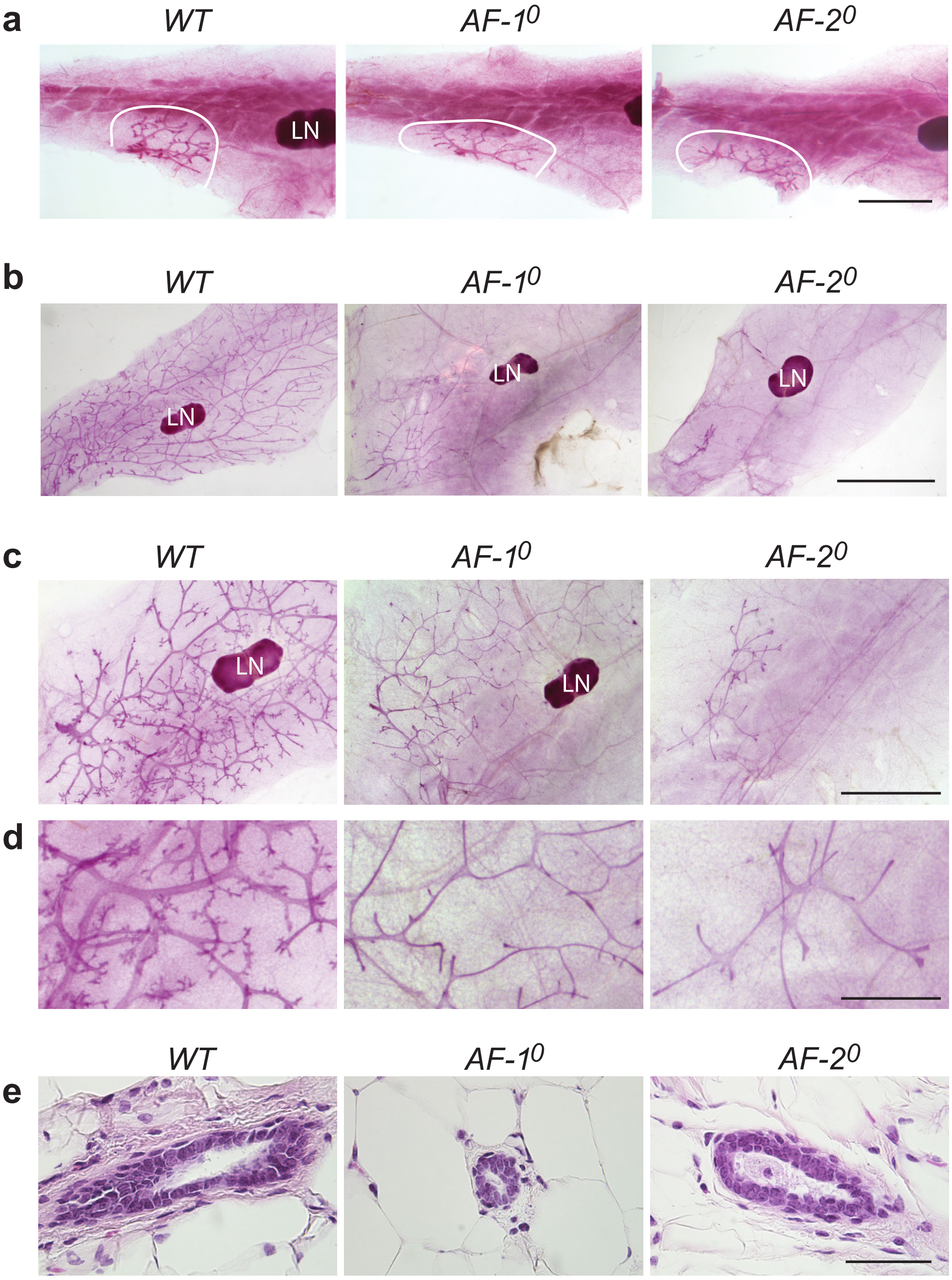

Supplementary Figure 1. Mammary gland phenotype of AF-1^0 and AF-2^0 mice.

a Stereo micrographs of mammary glands from 3-week-old *WT*, *AF-1^0* and *AF-2^0* females. Scale bars: 4 mm. **b** Stereo micrographs of adult, 8 to 12-week-old, *WT*, *AF-1^0* and *AF-2^0* females. Scale bars; 4 mm. **c** Stereo micrographs of adult (>13-week-old) *WT*, *ERαAF-1^0* and *ERαAF-2^0* females. Scale bars: 6 mm. **d** Higher magnification, scale bars: 2 mm. **e** H&E stained sections of mammary glands from 3-week-old *WT*, *AF-1^0*, and *AF-2^0* mice. Scale bar; 50 µm. LN: subiliac lymph node.

2

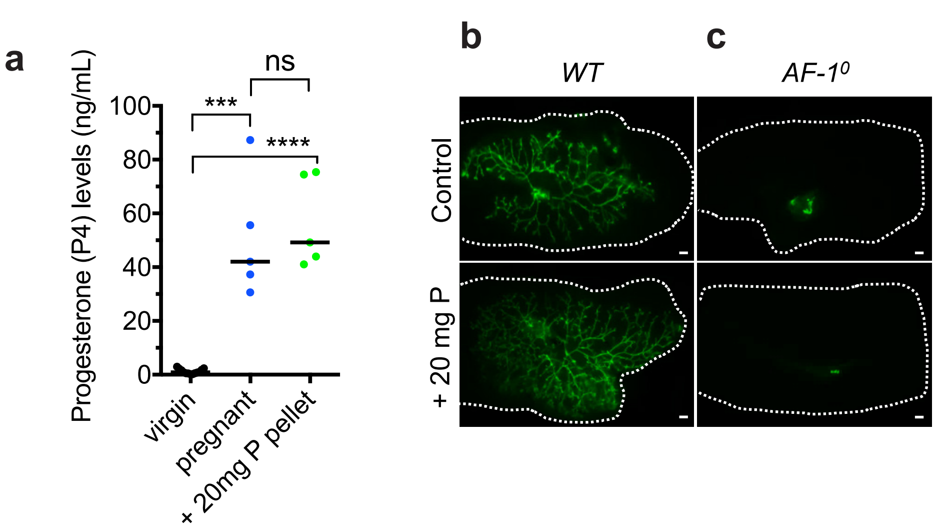

Supplementary Figure 2. Effect of *in vivo* progesterone exposure on AF-1[0] mammary epithelia.

a Dot plots showing plasma levels of progesterone determined by LC/MS in virgin, pregnant, and progesterone-treated animals (n=5-7). Horizontal lines depict median, Student's unpaired t-test, two-tailed. **b, c** Fluorescence stereomicrographs of contralateral inguinal mammary fat pads engrafted with mammary epithelium from *WT* and *AF-1[0]* littermates. Scale bar; 100 µm. Control and progesterone-treated recipients are shown.
